# Inclusive second level Religious Education in Ireland today: what do teachers say?

**DOI:** 10.1007/s40839-021-00144-8

**Published:** 2021-09-16

**Authors:** Amalee Meehan, Derek A. Laffan

**Affiliations:** National Anti-Bullying Research and Resource Centre (ABC), Institute of Education, Dublin City University, St Patrick′s Campus, Dublin 9, Ireland

**Keywords:** Religious Education, Teachers, Second level, Bullying, Catholic

## Abstract

The Irish religious landscape is changing. Census data reveal that the percentage of those who identify as Catholic is in steady decline, while the proportion of those with no religion continues to rise. Christian religious practice in Ireland is also decreasing, especially among young people. Catholic schools, once the dominant provider of second level education, are now in a minority. This changing landscape has influenced Religious Education in second level schools. It is now an optional subject, and the historic tradition of denominational, confessional Religious Education has given way to an approach designed to be inclusive of students of all faith and none. Yet the surrounding discourse is unsupported by the perspectives of Religious Education teachers. This study attempts to address this knowledge gap by investigating their views and experiences, particularly with regard to inclusion. Results indicate that teachers are concerned about ‘religious students’. Whereas new to the Irish context, this reflects international research which suggests that in a rapidly secularising society, those who continue to practise any faith, especially the once-majority faith, are vulnerable. Findings signpost evidence of this, with RE teachers most concerned about the bullying of Catholic students and least concerned about the bullying of atheists.

## Introduction

The shape of religious identity and practice in Ireland is changing. Census data indicate that the Catholic population has fallen by approximately 17% over the last five decades.^1^ At the same time, the proportion of those with no religion continues to rise. In 2016, 9.8% of the population identified as non-religious, up from 5.9% in 2011, and 0.04% in 1961 (Central Statistics Office, 2004, 2017). A recent study on religious affiliation among 16–29 year-olds in Ireland amplified this occurrence, with 58% of this age group identifying as Christian (54% Catholic, 2% Protestant, 2% other Christian), 1% Muslim, 3% other religions, and 39% not religious (Bullivant, 2018, p. 6). International patterns in Western, once Christian-majority nations, echo this trend towards no-religion (PEW Research Center, 2019).

Along with Catholic identity, Christian religious practice in Ireland is steadily decreasing, especially among young people. 26% of young people in the 16–29 age group say they never attend a religious service (Bullivant, 2018, p. 7). This trend is not particular to Ireland: since at least the beginning of this millennium, adolescents in England who regularly attend and participate in religious activities are a minority among their peers (Kay & Francis, 2001). On the other hand, it seems religious identity among second level students is still a feature: the most recent My World Survey found that 91% of this age group identifies with some religion (Dooley et al., 2019, p. 13).

These patterns and the increasing pluralism of secular and religious views among the population raise questions for the teaching and learning of all second level^2^ subjects including Religious Education (RE) such as: to what extent are teachers prepared for this religiously pluralistic environment; how inclusive is RE?

This article arises out of a research project funded by the Irish Human Rights and Equality Commission, which investigates views and experiences of teachers and minority belief students of Religious Education (RE) in second level schools in Ireland. Giving voice to RE teachers in order to understand their views and experiences, and ensuing implications for inclusive second level Religious Education, was a specific focus. The project was conducted by the National Anti-Bullying Centre (ABC) at Dublin City University (DCU). Most importantly for the ABC is the question of bullying: in this landscape can a young person’s religious identity/practice make them more or less vulnerable to bullying? Findings indicate that teachers are concerned about all ‘religious students’. This echoes the growing field of research which suggests that in a rapidly secularising society (cf. Norway, Sweden, UK) those who continue to practise any faith, especially the once-majority faith are vulnerable to bullying (cf. Ipgrave, 2012; Kittelmann Flensner, 2018; Moulin, 2016; Schihalejev et al., 2020). Findings seem to support this, with RE teachers most concerned about the bullying of practising Catholic students and least concerned about the bullying of atheists.

## Second level Religious Education in Ireland: the context

RE in state funded schools across Europe is the subject of much debate. Although the role and best approach to Religious Education are contested (cf. McKinney & McCluskey, 2017), the vast majority of European countries accept the necessity of school based Religious Education (Schreiner, 2013). Most European states provide Religious Education in publicly funded schools (NCCA, 2017, p. 25). In Scotland, England and Wales, for instance, a legal provision exists for RE as a core (compulsory) subject for all pupils (McKinney & McCluskey, 2017; Stuart-Buttle, 2017). Findings from a number of research projects across Europe agree that young people highly regard the place of RE and want a safe space to learn and talk about their own and others’ religions, beliefs and truth claims in schools (NCCA, 2017, p. 31).

Over the last two decades, in light of increasing social, cultural and religious tensions in many European countries, the Council of Europe has increasingly looked to RE as a means of promoting intercultural understanding and respect for diverse beliefs (NCCA, 2017, p. 28). More recently the Council’s recommendations go beyond just teaching about religion; they promote the development of attitudes such as sensitivity and respect for religious and non-religious traditions, as well as competencies such as religious literacy and understanding. According to the Council, such attitudes and competencies are necessary for intercultural living, and Religious Education has an important contribution to make in this regard.

### Second level Religious Education in Ireland today^3^

Up until the Education Act of 1998, the Irish state was effectively prohibited in involvement in second level RE. As a result, denominational school patrons filled the gap, leading to a system of denominational confessional RE (religious instruction). The Education Act (1998) removed this prohibition. Reflecting an international shift (Rymarz, 2012; Stuart-Buttle, 2017), the once dominant denominational and confessional tradition has since given way to an approach led by the state, designed to be inclusive of students of all faith and none.

The first state certified Junior Certificate Religious Education Syllabus (JCRES) was introduced in 2000, followed by the Leaving Certificate Religious Education Syllabus in 2003. Developed by the National Council of Curriculum and Assessment (NCCA) on behalf of the state, the JCRES encouraged students to reflect on human experience and to understand and interpret that experience. A significant aim, among others, was to foster an appreciation of ‘the richness of religious traditions and to acknowledge the non-religious interpretation of life’ (Department of Education & Science, 2000, p. 5). It also offered opportunities to ‘develop an informed and critical understanding of the Christian tradition’ (4).^4^ At the time the JCRES was introduced, the Irish Catholic bishops offered guidelines for how the syllabus might be used in Catholic schools and for the faith formation of Catholic students (Irish Catholic Bishops’ Conference, 1999).

#### The specification for Junior Cycle Religious Education (2019)

In line with the reform of Junior Cycle as outlined in the *Framework for Junior Cycle* (DES, 2015), a specification for Junior Cycle Religious Education (NCCA, 2019) has been incrementally implemented in schools since 2019. Religious Education, understood as the ‘critical encounter’ between religion and education (NCCA, 2017, p. 6), continues as a state-certified subject. Like its predecessor the JCRES, this specification is intended for all students, whatever their religious faith or worldview. It exposes students to a broad range of religious traditions and to the non-religious interpretation of life (NCCA, 2019, p. 4). It does not ‘provide religious instruction in any particular religious or faith tradition’ (DES, 2018, p. 2). Broad guidelines from the Irish Catholic Bishops entitled *Junior Cycle Religious Education in the Catholic School* are informed both by the *Framework* and accompanying documents, and by the specification (Council for Catechetics of the Irish Episcopal Conference, 2019).

### School patronage and RE

The school patron has a legal right and responsibility to uphold the characteristic spirit of the school (Government of Ireland, 1998). Religious Education can be one (among many) expressions of that characteristic spirit, giving patrons the right and responsibility to influence the approach to RE in their schools. Parents and children also have a right to expect RE in accordance with school ethos. In broad terms, patronage of second level schools falls into three sectors:

- Voluntary secondary schools, usually denominational but also including recent, non-religious patrons such as Educate Together.^5^ Schools with a Christian ethos fall into this category—just short of 50% of second level schools (see Table 1).
- Schools and community colleges managed on behalf of the state by local Educational and Training Boards (ETBs). ETBs, formerly Vocational Education Committees (VECs), are subcommittees of the Department of Education and Skills (DES). These schools are usually multi-denominational.
- Community and comprehensive schools, usually resulting from an amalgamation of schools. In these cases, the state (through the DES) and another body (usually a religious congregation or local bishop who had been patron of an amalgamating school) act as co-patrons. These are also usually multi-denominational.

**Table 1:** Number of post primary schools by sector 2019/2020

School type	Frequency	Percentage
Voluntary secondary Christian	354	49
Voluntary secondary secular	19	2.6
Voluntary secondary (An Fóras Patrúnachta)	6	0.8
Vocational (ETB)	246	34
Community and comprehensive	96	13
Total	723	100

*Sources* https://www.education.ie/en/Publications/Statistics/Key-Statistics/key-statistics-2019-2020.pdf; https://www.educatetogether.ie/schools/find-a-school; http://www.foras.ie/en/scoileanna; https://www.schooldays.ie/secondary-schools-in-ireland/Church-of-Ireland

In recent years, the Department of Education and Skills has made clear the required approach to RE in community and ETB second level schools. The Department does not require any school to include Religious Education at Junior Cycle as a mandatory subject. Accordingly, schools ‘have discretion to determine if they provide the subject at all or if it is to be mandatory or optional in any particular class group or year’ (DES, 2018, p. 2). Further, the more formative/confessional approach to RE as a subject is explicitly dealt with. Where ‘religious instruction and worship in accordance with the rites and practices of a particular denomination’ (DES, 2018, p. 3) is offered in ETB schools or community schools and colleges:

- It must not be associated in any degree with the NCCA developed syllabus/specification
- It must not be provided in timetabled class periods
- A newly required opt-in by parents for their children is necessary (DES, 2018).

#### RE in Catholic schools

Byrne (2021) explains that the Irish Catholic Bishops are committed to RE in their schools and to the developments in state sponsored RE since the JCRES of 2000. They recognise the need for a Religious Education that opens students to different religious perspectives. ‘Everyone is asked to bring their beliefs and values, their very selves, into the Religious Education classroom and to open their mind and heart to the deepest meaning of life’ (Byrne, 2021, pp. 8–9).

The Irish Catholic Bishops’ response to the *Framework for Junior Cycle* (2015) upholds Religious Education as a manifestation of school ethos. It explains the importance of RE in an holistic education, the need for a realistic understanding of the needs of young people and ‘the opportunities and challenges they face in the secular world that dominates their lives and the continuing willingness of the Catholic faith community to put its best resources at the disposal of the young’ (Irish Catholic Bishops’ Conference, 2017, p. 5).

## Methodology

The research was completed in 2019 and involved an online survey of 214 Religious Education teachers. Ethical approval was obtained from the Dublin City University research ethics committee. The online survey was issued to all second level schools in the Republic of Ireland and circulated to RE teachers through school principals. Principals were invited to share a link to the survey with the RE teachers in their schools. Participants came from a cross section of second level schools in Ireland, with schools from all three patronage sectors represented.

The data from the online survey were transferred to an Excel spreadsheet and statistically analysed. Statistical Package for the Social Sciences (SPSS) in conjunction with Survey Monkey analytical software and Microsoft Office software data were used as an analytical tool. A thematic approach was used to analyse comment box responses.

### Participant profile

214 RE teachers participated in the study. The majority identified as Roman Catholic (85%) with 2% Church of Ireland, 5% multiple religious beliefs and 4% no religion. 66% described their religious beliefs as very important to them. 80% of participants identified as female, 20% as male, reflecting the overall gender gap in the teaching profession in Ireland (DES, 2021).

## Findings: what RE teachers had to say

When it came to the place and purpose of Religious Education on the curriculum, 67% of participants felt that RE should be a state examined subject in second level schools. 64% said that RE should be a mandatory subject in both Junior and Senior Cycle. With subject matter extending to all world religions and the non-religious worldview, teachers felt it is inclusive of all students and agreed that all beliefs should be respected and accommodated in RE.

**Fig. 1:**
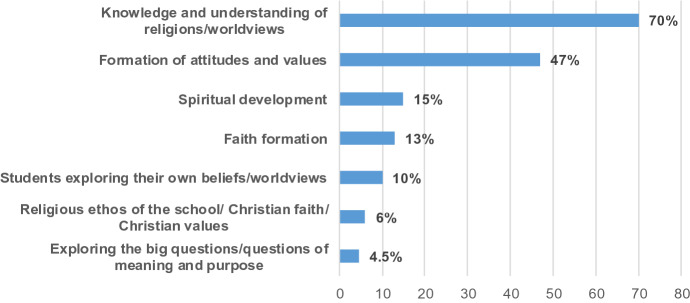
Reported main goals of Religious Education among RE teachers

There was quite a variation in how teachers described their main goal in teaching RE. Approximately one third of respondents listed a single main goal such as ‘exam results’, ‘acceptance and tolerance of other faiths and none’, or ‘teach the syllabus to all students’. The remainder offered compound responses (as reflected in Fig. 1), i.e. responses containing more than one goal, such as

- “To broaden student knowledge of world religions with the aim of promoting tolerance and respect. I always want students to reflect on their own spirituality, whatever that may be.”
- “To inform students about different world views, equip them with skills to reflect[ion] on their own and promote respect for diversity.”
- “To inform students about faith and to encourage them to question faith and develop their own view of what faith is and how best it will serve them.”
- “To inform students about the role religion has played in the lives of people past and present (by following the JC syllabus). To also instil in my students, a place to develop their spirituality if they so wish.”
- “To help all students learn about, experience and enjoy learning about ALL world religions while keeping the ethos of Nano Nagle, the Presentation Order & Christianity visible in our school.”
- “Education about religions and their elements and beliefs and rituals, not faith formation.”

These intersecting goals are not at odds with each other; rather they reflect the multidimensional aims of second level RE.

Consistent across all three sectors, 70% of responses highlighted knowledge and understanding of religions/worldviews as a main goal of teaching RE. Formation of attitudes and values was a main goal of approximately half of teachers, with tolerance, respect and acceptance the most commonly cited. 13% of responses referred to faith formation, well below the proportion of schools with a Christian ethos participating in this study (57%). Other, less frequently cited, main goals in teaching RE included spiritual development, students exploring their own beliefs/worldviews, the religious ethos of the school/the Christian faith/appreciation of Christian values, and exploring the big questions/questions of meaning and purpose (see Fig. 1).

### Preparedness for teaching minority faith and non-religious pupils

The vast majority of participants (83%) said they emphasize the diversity of religious/non-religious views in most classes/every class. Interestingly, whereas students were interested in learning about world religions, morality, and social issues, some teachers said that it was getting harder to motivate their students to study Catholic RE.

When assessing their preparedness for teaching minority faith pupils, an average of 11% felt not prepared (see Table 2). This figure fell to 9% when it came to teaching non-religious students (see Table 3). Across sectors, the majority felt *somewhat* prepared to teach minority/non-religious students, suggesting a need for ongoing, quality CPD for RE teachers in this area.

**Table 2:** Preparedness for teaching minority faith students by school type

	Very prepared	Somewhat prepared	Not prepared
Voluntary secondary school	40 (33%)	65 (54%)	15 (13%)
ETB school or community college	21 (35%)	33 (55%)	6 (10%)
Community or comprehensive school	17 (55%)	11 (35%)	3 (10%)

**Table 3:** Preparedness for teaching non-religious students by school type

	Very prepared	Somewhat prepared	Not prepared
Voluntary secondary	50 (42%)	59 (49%)	11 (9%)
ETB school or community college	26 (43%)	31 (51%)	4 (7%)
Community or comprehensive school	19 (61%)	9 (29%)	3 (10%)

### Provisions for students of minority/non-religious beliefs during RE

When asked about provisions made for students of minority/non-religious beliefs during RE, a majority responded that all students were encouraged to participate and to share their views regardless of their religious beliefs or faith background. Most teachers also referenced the choice to opt-out. Findings indicate that opting out of RE takes different forms in different schools: this appears to depend upon resources, the RE programme, history of requests/numbers making the request, and specific policy/ethos led arrangements. In some schools, students who opt out could go to a supervised study area such as the library or study hall. That was clearly not an option in other schools due to a lack of resources such as space/personnel to supervise students. In a small number of cases, teachers were expected to come up with their own arrangements. The array of opt-out practices in schools for students of minority/non-religious beliefs include:

- “They remain in class and can read only, cannot do study or homework to prevent educational advantage.”
- “They sit in the class but do not have to participate.”
- “They sit at back of class and make a contribution if they wish.”
- “They are welcome to join if they choose; otherwise, they go to the study hall.”
- “Any student who chooses to opt out sits at the back of the room, studying their own faith.”

The opt-out facility is intended to promote the student’s agency where religious identity is concerned. However, teachers articulated the unsatisfactory nature of studying alone while the rest of the class continued with the RE lesson. They suggested that this might be addressed through curricular changes and additional resources. Since this study, curricular changes have been incrementally introduced with the Junior Cycle RE specification (2019); these changes suggest that the opt-out is no longer necessary in ETB, Community or Comprehensive schools (DES, 2018).

### Religious/belief based bullying

For 88% of respondents, religious based bullying either was not an issue in their schools, or they were not aware of it, with comments such as “I haven’t heard of any bullying based on religious belief in the school.” However, a number of teachers expressed concern. One stated “There can be hostility from non religious students towards students who express faith at times.” Another said that “Strong beliefs by students can be ridiculed.” Teachers singled out Christians as the most vulnerable group: “expressing religious based convictions can lead to low level bullying by staff members…e.g. expressing anti-abortion views.” Another commented “I suspect Christians get the greatest flak today. There is a general intolerance of the Christian worldview which needs [to be] addressed.” Whereas new to Ireland, this concern echoes similar findings in other secularising nations where Christianity once dominated such as Australia, the United Kingdom, Sweden and Estonia (cf. Schihalejev et al., 2020).

Comments such as “many students profess no active faith. Few students express active participation in their faith” are interesting in the context of the My World Survey (Dooley et al., 2019), which found that 91% of this age group identify as religious. It seems that young people identify as religious at some level, but may find it difficult to appear so. As one teacher suggested: ‘holding a religious worldview can be a lonely experience in modern Ireland.’

### Negative stereotyping

When it comes to negative stereotyping of students, teachers are most concerned about those who identify as Catholic (12%) and least concerned about negative stereotyping of those who identify as atheist (2%). Of the respondents who explained their answers, 50% voiced concern about anti-religious sentiment/behaviour such as ‘the lazy way that Muslims can be categorised as terrorists, and Catholics as paedophiles or supportive of such behaviour’.

Students who identify as Catholic were most frequently the subject of concern. 33% of those who voiced concern singled out Catholicism/Catholics with comments such as:

- ‘A Catholic student is more likely to be ridiculed or laughed at for their faith position so they tend to be silenced by the prevailing trend towards a secular humanist worldview.’
- ‘It is now seen as archaic to hold Catholic values among the student body.’
- ‘It is socially acceptable in Ireland to insult/belittle Catholics/Catholicism.’

### Findings from the open forum

One aim of this study was to give RE teachers an opportunity to raise issues of relevance to them i.e. to identify what they feel are the dominant opportunities and/or concerns for inclusive RE today. Therefore, the survey ended with an open forum style question: *The purpose of this survey is to assist in providing guidelines for inclusive RE... please add your voice here.* Of the 214 teachers who filled in the survey, 118 chose to participate in this open forum. Three main themes emerge from the data as follows.

1. The most dominant theme was the importance of Religious Education as a subject on the curriculum. The rationale for this was twofold: it can prepare young people to live in a global society and it contributes to the spiritual/moral development of students. Both echo the rationale of the JCRES (2000) and the JCRES (2019).
2. Some respondents (15%) were concerned about Catholic school ethos and/or about the effects of eroding Catholic ethos. They spoke of ‘having to apologise for being Catholic’ and ‘having to justify a Catholic ethos.’ Teachers talked about the negative view of faith schools—how they are portrayed in the Irish media and depicted in Irish society. They felt that this is inaccurate and does a disservice to society, that faith schools have an important role to play and should be allowed to fulfil that role, and that the secular/non-religious agenda can often be promoted instead of one that is fully inclusive. Some participants expanded this view to faith in general and the importance of faith to individuals and to a truly pluralist community.
3. A third theme (14% of respondents) was that students of faith are vulnerable to bullying. Some suggested that the negative view of faith and faith schools contributes to this effect, making students of faith a vulnerable group. The most vulnerable group of second level school students to emerge from the open forum are practising Catholics; the least vulnerable are those who profess a non-religious or atheist worldview. Teachers see evidence of pressure to be/identify as a non-believer.

## Discussion

Overall, RE teachers are positive about the provision of RE in second level schools and agree that it should continue to be provided. Although the main goal might differ, there is much support for RE as a subject which provides significant opportunities to promote tolerance and understanding between faiths and with the non-religious view. Most teachers see RE as primarily an academic subject, inclusive of all world religions and the non-religious worldview. The variety of goals they articulate reflect the broad aims of RE at second level in Ireland and the enlarging view of the role and relevance of RE across Europe. Although all aims of RE (DES, 2003; NCCA, 2019) emerge from the survey, some are more dominant than others. Knowledge/understanding of religious traditions and the non-religious interpretation of life, and formation of attitudes such as tolerance and respect, constitute the main goals of teaching. This resonates with European practice and the view of the Council of Europe outlined earlier. A minority of 13% of responses refer to the role of RE in faith formation, well below the proportion of schools with a Christian ethos participating in this study (57%). This may be of significance to patron bodies. Mostly drawn from the voluntary sector, the majority of these position faith formation as one goal among others. For instance, one teacher describes how “[we] deliver the curriculum as we follow the junior cert religion course. We also provide opportunities for students to partake in services/reflection following the liturgical calendar.” Finally, a small minority identify as a main goal students exploring their own beliefs/worldviews, and engaging with the big questions of life. This may change with the emphasis on ‘Exploring Questions’ in the specification for Junior Cycle RE (2019).

Teachers’ views on the place and purpose of RE are interesting in the context of the effects of the Covid-19 pandemic. Research during this period indicates that many people see themselves as having become more reflective, more prayerful and closer to God during the more severe period of lockdown. One result of Covid-19 is that people seem to be asking: What is really important? What gives us meaning and purpose? Where should we root our values (Byrne & Sweetman, 2020)? Although young people value RE and want a safe space to learn and talk about their own and others’ religions, beliefs and truth claims in schools (NCCA, 2017), opportunities for them to talk about religion or faith outside the classroom are limited (Cullen, 2019, p. 280). RE should be significant in allowing young people to engage with the spiritual and religious questions they may not be addressing elsewhere. However, the opt out arrangement, intended to promote student agency, appears inconsistently applied and often unsatisfactory. It is important to remember that this data pertains to the period before the implementation of the JCRE specification from 2019; the opt-out is no longer deemed necessary in schools under sole or joint state patronage (DES, 2018).

The teachers’ concerns around the vulnerability of religious students reflect the growing field of international research suggesting that in rapidly secularising societies, those who continue to practise any religion are vulnerable to bullying, especially the previous majority religion (cf. Ipgrave, 2012; Kittelmann Flensner, 2018; Moulin, 2016; Schihalejev et al., 2020). For instance, Ipgrave identified that when an atheist cool sweeps the school, adolescents can consider religious participation as ‘abnormal’, with adverse consequences for young people who practise. In these settings, religious adolescents risk ridicule and social exclusion (Ipgrave, 2012). Similarly, the international research project REDCo (Religion in Education. A Contribution to Dialogue or a Factor of Conflict in Transforming Societies of European Countries) undertaken with 14–16 year olds found that some religiously-committed students feel vulnerable in the classroom (Weisse, 2011). Ipgrave (2012) concludes that when students feel forced to conceal or deny their religious identity, both personal and communal (school community) wellbeing are compromised. This has implications for Irish schools, where it seems that many young people are religious at some level, but may not want to appear so.

## Conclusion

The move away from Catholic-focused RE towards an approach that is inclusive of all religions and the non-religious perspective is enshrined in the specification for Junior Cycle RE (2019). A follow up survey of RE teachers within 3–5 years to assess the impact of the specification would be useful. With many RE teachers feeling somewhat (but not very) prepared to teach minority/non-religious students, ongoing quality Continuing Professional Development in this area is recommended.

It seems that all students who practise a religion can experience problems in school. In this context, teachers had specific concerns about students who were practising Catholics being targeted for bullying. However, some teachers consider this to be bigger than schools; if it is an issue of wider society, it cannot be left to schools to deal with alone. The experiences of those from a traditionally majority position which goes into rapid decline are a subject of some concern internationally. There was evidence from this study to support the existence of this concern in the Irish context. Given the personal and communal wellbeing implications, this needs to be taken seriously, investigated further and addressed.

Finally, the findings presented in this report only pertains to the perspectives of RE teachers. The voices of both students and teachers who practise a religious faith would add to this field of research, so that the experience of being religious in a school setting can be more deeply and broadly explored.

## Footnotes

1 In 1961 the percentage of the population who identified as Catholic was 94.9, the highest recorded. 78.3% of the population identified as Catholic in 2016, the lowest recorded. Source https://www.cso.ie/en/releasesandpublications/ep/p-cp8iter/p8iter/p8rrc/.

2 Second level (also known as post-primary) in the Irish context refers to secondary/high school.

3 In Ireland, students aged 12–15 approximately follow the Junior Cycle during the first 3 years of second level school. Students then go on to Senior Cycle, which takes 2 or 3 years, depending on an optional Transition Year. The Leaving Certificate is the high stakes, state certified, terminal examination which takes place at the end of Senior Cycle.

4 These aims also apply to the *Leaving Certificate Religious Education Syllabus* (2003).

5 There is one voluntary secondary school in Ireland with a Jewish patron. Stratford College was founded by the Dublin Jewish Community and provides second level education within a Jewish ethos. The largest non-Christian minority in Ireland is the Muslim population at 1.3%. The Islamic Foundation of Ireland is patron of two Muslim national schools and is seeking to become patron at second level. At present there is no Islamic post primary school in Ireland.
